# Simultaneously Achieving Highly Efficient and Stable Polymer:Non‐Fullerene Solar Cells Enabled By Molecular Structure Optimization and Surface Passivation

**DOI:** 10.1002/advs.202104588

**Published:** 2022-01-15

**Authors:** Bowen Liu, Xiao Su, Yi Lin, Zerui Li, Lingpeng Yan, Yunfei Han, Qun Luo, Jin Fang, Shangfeng Yang, Hongwei Tan, Chang‐Qi Ma

**Affiliations:** ^1^ School of Nano‐Tech and Nano‐Bionics University of Science and Technology of China Hefei 230026 P. R. China; ^2^ i‐Lab & Printable Electronics Research Center Suzhou Institute of Nano‐Tech and Nano‐Bionics Chinese Academy of Sciences Ruoshui Road 398, SEID, SIP Suzhou 215123 P. R. China; ^3^ College of Chemistry Beijing Normal University Xinjiekouwai St. Beijing 100875 P. R. China; ^4^ Department of Chemistry Xi'an Jiaotong Liverpool University Renai Road 11, SEID, SIP Suzhou 215123 P. R. China; ^5^ CAS Key Laboratory of Materials for Energy Conversion Department of Materials Science and Engineering University of Science and Technology of China Hefei 230026 P. R. China

**Keywords:** degradation and stability, interfacial photon decomposition, non‐fullerene acceptor, polymer solar cells, structure‐property relationship

## Abstract

Despite the tremendous efforts in developing non‐fullerene acceptor (NFA) for polymer solar cells (PSCs), only few researches are done on studying the NFA molecular structure dependent stability of PSCs, and long‐term stable PSCs are only reported for the cells with low efficiency. Herein, the authors compare the stability of inverted PM6:NFA solar cells using ITIC, IT‐4F, Y6, and N3 as the NFA, and a decay rate order of IT‐4F > Y6 ≈ N3 > ITIC is measured. Quantum chemical calculations reveal that fluorine substitution weakens the C═C bond and enhances the interaction between NFA and ZnO, whereas the *β*‐alkyl chains on the thiophene unit next to the C═C linker blocks the attacking of hydroxyl radicals onto the C═C bonds. Knowing this, the authors choose a bulky alkyl side chain containing molecule (named L8‐BO) as the acceptor, which shows slower photo bleaching and performance decay rates. A combination of ZnO surface passivation with phenylethanethiol (PET) yields a high efficiency of 17% and an estimated long *T*_80_ and *T*s_80_ of 5140 and 6170 h, respectively. The results indicate functionalization of the *β*‐position of the thiophene unit is an effective way to improve device stability of the NFA.

## Graphical abstract

Fluorine substitution increases the interaction with ZnO and accelerates the photon decomposition of A‐D‐A type (nonfullerene acceptor) NFA, while he *β*‐alkyl chains on the thiophene unit next to the C═C linker improves the stability of acceptor molecules by forming protecting atomic cage. When using PET‐treated ZnO and L8‐BO as the electron acceptor, *T*_80_ was estimated over 5000 h.

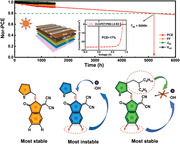

## 1 Introduction

**Figure 1:**
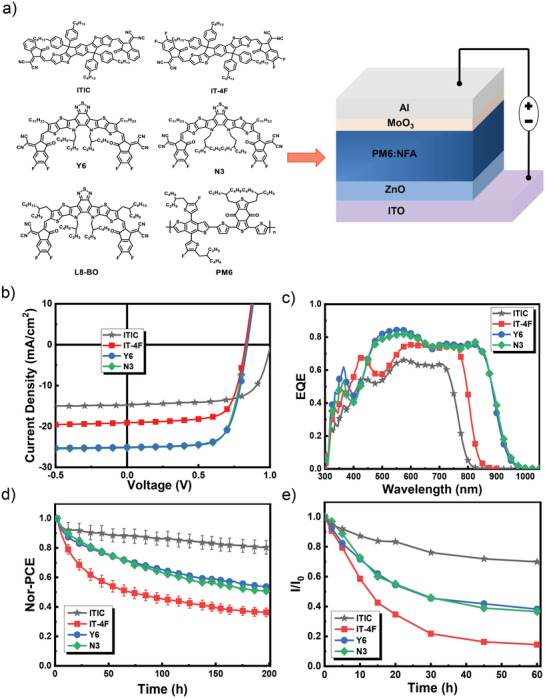
a) Molecule structures of active layer materials and device structure of solar cells studied; b) *J*–*V* curves and c) EQE spectra of PM6:NFAs cells; d) PCE decay curves of PM6:NFAs cells under white light illumination; and e) photon bleaching of the NFAs on ZnO surface.

With the advantages of being light‐weight, flexibility, and solution processability, polymer solar cells (PSC) have received widespread attention and become a promising new generation of solar cells.^[^ ^1^ ^]^ During the past few years, various high‐performance polymer donors^[^ ^2^ ^]^ and non‐fullerene acceptors (NFAs) have been developed,^[^ ^3^ ^]^ and power conversion efficiency (PCE) of PSCs have reached 18% rapidly.^[^ ^4^ ^]^ In fact, the breakthrough of A‐D‐A type non‐fullerene acceptors are the key to the leap in device PCE.^[^ ^5^ ^]^ Through donor−acceptor structure modifications,^[^ ^6^ ^]^ functional group substitution,^[^ ^5c^ ^]^ and side‐chain engineering,^[^ ^7^ ^]^ various high‐performance NFAs were developed and studied. DC‐IDT2T was first reported by Zhan et al. as the A‐D‐A type NFA in PSC, where the indacenodithiophene (IDT) unit was introduced as the *π*‐conjugated central donor moiety and 1,1‐dicyanomethylene‐3‐indanone as the terminal acceptor unit (Scheme S1, Supporting Information).^[^ ^8^ ^]^ Fusing IDT with two thiophene at both sides yielded a new acceptor molecule ITIC (**Figure** **1**a).^[^ ^9^ ^]^ By introducing two fluorine atoms on the benzene ring of 1,1‐dicyanomethylene‐3‐indanone unit, Hou et al. reported a new NFA IT‐4F (Figure 1a).^[^ ^5c^ ^]^ The smaller optical band gap of IT‐4F is beneficial for PSC since this increases the light harvesting ability of the blend,^[^ ^10^ ^]^ making IT‐4F a mostly widely used NFA in PSC. In 2019, Zou et al. synthesized a new acceptor molecule Y6, which has a ladder‐type dithienothiophen[3.2‐*b*]‐pyrrolobenzothiadiazole core unit fused with a benzothiadiazole (BT).^[^ ^11^ ^]^ Owing to the electron deficient nature of the BT core unit, Y6 has low lying HOMO/LUMO energy levels, and a high PCE of over 15% was achieved. By replacing the 2‐ethylhexyl with 3‐ethylheptyl on the pyrrole motif of Y6, Yan et al. synthesized a new acceptor N3,^[^ ^12^ ^]^ which has improved solubility and the consequently PCE in PSC. Very recently, functionalization of Y6 on the *β*‐position of the thiophene unit next to the C═C bond was performed and high performance NFA, including Y6‐1O by attaching one alkoxy chain on the Y6,^[^ ^13^ ^]^ L8 series,^[^ ^4c^ ^]^ with which a high PCE of over 18% were reported.

With the rapid development of PCE of PSC, stability of PSCs also received much attention in the last few years. In this aspect, polymer:fullerene solar cells were well investigated and different degradation pathways including crystallization of fullerene molecules,^[^ ^14^ ^]^ photon dimerization of fullerene molecules,^[^ ^15^ ^]^ interfacial photochemical reaction of polymer with MoO_3_,^[^ ^16^ ^]^ as well as nanomorphology changes of the photoactive layer under operation^[^ ^17^ ^]^ were clarified, and methods to improve the stability of the polymer:fullerene solar cells were developed.^[^ ^18^ ^]^ As for the NFA molecules, especially for the A‐D‐A type NFAs that have a C═C linker between the donor core and the accepter end group, more efforts were made on the understanding of the intrinsic stability of NFA molecules at different conditions. For example, Zhou et al. reported that the A‐D‐A type NFA molecules can react with polyethylenimine (PEI), yielding the disruption of the exocyclic C═C linker.^[^ ^19^ ^]^ Kim et al. compared the photon stability of IDTBR and IDFBR in air, and revealed that the IDFBR is more prone to degradation owing to the twisted *π*‐conjugated core unit that initiates fast photooxidation of the IDFBR.^[^ ^20^ ^]^ Du et al. compared the stability of ITIC derivatives with different substitutions (fluorine atom, methyl group) on the terminal acceptor unit, and results demonstrated that fluorination of the end‐group stabilizes molecules against light soaking, while methyl groups show an opposite trend.^[^ ^21^ ^]^ Koster et al. found that stability of the ITIC derivatives based cells is both depended on the NFA molecular structure as well as the device structure, that is fluorine substitution accelerates degradation in conventional device, while methyl substitution slows down degradation in the same device structure.^[^ ^22^ ^]^ All these reports provide a qualitative understanding of the stability of NFA molecules and the corresponding solar cells. However, there is a lack of deep insight of detailed degradation mechanism of the NFA solar cells and the specific influence of molecular structure on these degradation processes.

Zhou et al. first reported that IT‐4F tend to decompose on the ZnO surface under UV light illumination, which is originally due to the photocatalytic activity of ZnO and consequently leads to the poor performance stability of IT‐4F based cells under light illumination.^[^ ^23^ ^]^ Park and Son demonstrated also that ITIC underwent interfacial photon decomposition on ZnO surface, making the ITIC based cell less stable than fullerene‐based cells.^[^ ^24^ ^]^ By comparing the stability difference of the IT‐4F cells with different ZnO interlayer, we recently proved that photon generated hydroxyl radicals on the ZnO surface is the chemical reactive species that causes the breaking of the C═C bonds.^[^ ^25^ ^]^ With the better understanding on the detailed degradation mechanism of the NFA cells, methods to improve device stability, including blending with fullerene molecules,^[^ ^26^ ^]^surface treatment with Lewis acids,^[^ ^27^ ^]^ a thin PEI layer,^[^ ^28^ ^]^ or a self‐assembled monolayer^[^ ^29^ ^]^ were reported, supporting the proposed degradation mechanism. Despite these excellent research works in improving the stability of the cells, these cells showed lower initial efficiencies less than the optimized cell performance, and most of the cells showed a PCE lower than 15%.

To create a guideline for choosing a better NFA molecule for PSCs, in this paper, we systematically investigate the molecular structure–stability relationship of NFA molecules. We compared the performance decay rates of the ITIC, IT‐4F, Y6, and N3 based cells and correlated the results to the photobleaching processes on the ZnO surface. And the effect of fluorine substitution (ITIC versus IT‐4F), the core electron donor structure (IT‐4F versus Y6/N3), as well as the side‐chain substitution (Y6 versus N3) on the device stability were clearly clarified. DFT calculations and NMR measurements were performed to fully understand how the molecular structure influences the interaction between ZnO and NFA molecules. With this useful guideline, we choose the L8‐BO, which has a 2‐butyloctyl chain on the *β*‐position of the thiophene ring as the electron acceptor in the fabrication of PSCs. In combination with the phenylethanethiol‐treated ZnO layer, a L8‐BO cell with PCE over 17% and long *T*_80_ and *T*s_80_ of 5140 and 6170 h were achieved, demonstrating a successful way to simultaneously achieving high PCE and high device stability.

## 2 Results and Discussion

### 2.1 Performance and Decay Dynamics of Inverted Polymer:NFA Solar Cells

To understand the influence of NFA on the degradation processes of the PSC, we fabricated and tested the PSCs with an inverted structure of ITO/ZnO/PM6:NFA/MoO_3_/Al (Figure 1a). Four A‐D‐A type small molecular NFAs were studied first, including ITIC, IT‐4F, Y6, and N3. The current density–voltage (*J*–*V*) characteristics and external quantum efficiency spectra (EQE) of the PSCs under simulated AM 1.5G solar illumination are shown in Figures 1b and 1c, respectively. The photovoltaic performance data are listed in **Table** **1**. As seen here, the ITIC and IT‐4F based cells show the highest PCE of 10–11%,^[^ ^30^ ^]^ whereas the Y6 and N3 based cells showed a highest PCE of 15%,^[^ ^11^, ^12^, ^31^ ^]^ which are comparable to the literature reported PCE for the corresponding cell with the same inverted device structure. All these cells were then aged at the maximum power point (mpp) inside the glovebox with continuous white light illumination. Figure 1d depicts the PCE decay curves of these cells over 200 h. As seen here, the IT‐4F based cells under light illumination were reduced to 36% of their initial values after aging, while the PCEs of the ITIC based cells still maintained 80% of their initial values over the same aging period. Interestingly, the Y6 and N3 based cells have similar decay traces and keep ≈50% of their initial values. To quantitatively analyze the degradation processes of cells, the PCE decays were numerically fitted to a stretched exponential model according to Equation (1):^[^ ^32^ ^]^

(1) $$PCE\left(t\right)=PCE\left(\infty \right)+\alpha \times \exp{\left(-\frac{t}{\tau }\right)}^{\beta }$$

where *τ*, *α*, and PCE(∞) represent the mean lifetime, pre‐exponential factor (degradation amplitudes), and the intercept (the saturated PCE over a long time aging), respectively. The stretching exponent *β* is in the range 0 < *β* ≤ 1, which indicates the complexity of the decay process. The fitting decay curves are shown in Figure S1, Supporting Information, and the corresponding parameters are listed in Table 1. As seen here, the mean lifetimes for ITIC, IT‐4F, Y6, and N3 cells are 539, 41, 150, and 148 h, respectively, indicating a performance decay rate of the solar cells as IT‐4F > Y6 ≈ N3 > ITIC. Since all these cells have the same donor (PM6) but different NFAs, the different decay rate for the cells under the same aging condition suggests that the performance decay of the cells is directly related to the molecular structure of acceptors.

**Table 1:** Performance of devices based on PM6 with different NFA

Acceptor^a)^	*V*_OC_ [V]	*J*_SC_ [mA cm^−2^]	FF	PCE [%]	PCE_max_ [%]^d)^	*τ* [h]	*α*	PCE(∞)	*β*
ITIC^b)^	1.00 ± 0.001	15.06 ± 0.086	0.67 ± 0.007	10.14 ± 0.127	10.28	539	0.48	0.53	0.54
IT‐4F^b)^	0.83 ± 0.003	19.12 ± 0.025	0.70 ± 0.004	11.15 ± 0.018	11.18	41	0.73	0.30	0.65
Y6^c)^	0.85 ± 0.003	25.12 ± 0.144	0.70 ± 0.003	14.96 ± 0.073	15.03	150	0.69	0.31	0.76
N3^c)^	0.84 ± 0.001	25.06 ± 0.251	0.71 ± 0.006	14.93 ± 0.054	15.00	148	0.69	0.30	0.77

^a)^ Device structure ITO/ZnO/PM6:NFA/MoO_3_/Al, cell area 0.09 cm^2^, averaged device performance over eight individual cells;

^b)^ Blend ratio PM6:NFA (1:1 in weight);

^c)^ Blend ratio PM6:NFA (1.0:1.2 in weight);

^d)^ PCE of the best cell.

### 2.2 Interfacial Photon Decomposition of NFAs on ZnO Surface

The comprehensive comparison of the decays of open‐circuit voltage (*V*_OC_), short circuit current (*J*_SC_), fill factor (FF), and PCE (see Figure S2, Supporting Information, for all the decay curves) showed that, for all these four different types of cells, *V*_OC_ and FF are rather stable over aging, while *J_SC_* decays much faster and dominates the overall performance decay of the cells. This is similar to our previous finding for the PM6:IT‐4F cell,^[^ ^25^ ^]^ indicating that the PM6:NFA cells might have a similar degradation mechanism, that is, the decomposition of NFA on the ZnO surface by the photon generated hydroxide radicals. To prove this, we measured the absorption spectrum changes of the ITIC, IT‐4F, Y6, and N3 films (≈10 nm) aged under LED white light. Figure S3, Supporting Information, shows spectrum changes of the film over aging. As seen there, the absorption of ITIC/IT‐4F component (600–750 nm)^[^ ^24^ ^]^ and Y6**/**N3 component (600–950 nm)^[^ ^11^, ^12^ ^]^ decreases gradually with the increase in light‐soaking time, suggesting that these acceptor molecules decomposed gradually on the ZnO surface. Figure 1e compares the decrease of the absorbance of these NFAs on the ZnO surface. Interestingly, the photobleaching rate of these NFAs on ZnO surface was found to be consistent with the decay rate of these NFA solar cells, confirming that photon decomposition of the NFA on ZnO surface is the main reason for the performance decay of the cells, and the stability of the NFA molecules directly determines the stability of the cells.

### 2.3 Product Analysis of the Photobleached Films

**Figure 2:**
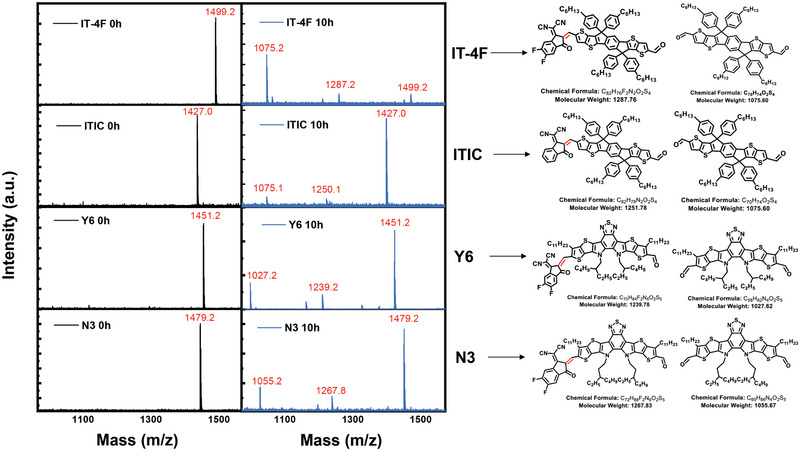
MALDI‐MS spectra of the pristine and aged acceptors on ZnO surface. The right side chemical structure showed the decomposed product.

To verify the detailed photochemical reaction at the ZnO/NFA interface, the products of the ZnO/NFAs films after light illumination were measured by matrix‐assisted laser desorption/ionization time‐of‐flight mass spectrometry (MALDI‐TOF‐MS). **Figure** **2** shows the MS spectrometry of the initial NFAs and the products of the films after being illuminated for 10 h. The initial ITIC, IT‐4F, Y6, and N3 samples showed an MS peak at m/z of 1427.0, 1499.2, 1451.2, and 1479.8 corresponding to the M+H^+^ of the NFA molecule. After light illumination for 10 h, decomposition of IT‐4F to mono‐ and bis‐aldehyde products with m/z of 1287.2 and 1075.2 were measured, confirming the breaking of the exocyclic C═C bond of NFA molecules. Similarly, m/z peak sets of 1250.1/1075.2, 1239.2/1027.2, and 1267.8/1055.2 were measured for ITIC, Y6, and N3, which correspond to the mono‐ and bis‐aldehyde products as well, confirming the decoupling of the core–donor and terminal acceptor moieties for all these three compounds during aging. For the aged IT‐4F sample, the M+H^+^ signal is very weak, while the m/z signal of 1075.2 is the most intensive one, indicating a large percent of decomposition of IT‐4F. In contrast, weaker m/z signals were measured for the ITIC, Y6, and N3 samples. These results are in good accordance with the ultraviolet–visible (UV–vis) absorption decay dynamic results (Figure 1e), confirming that breaking of the exocyclic C═C bond under light illumination is the detailed decomposition reaction.

### 2.4 Molecular Structure–Performance Relationship

**Figure 3:**
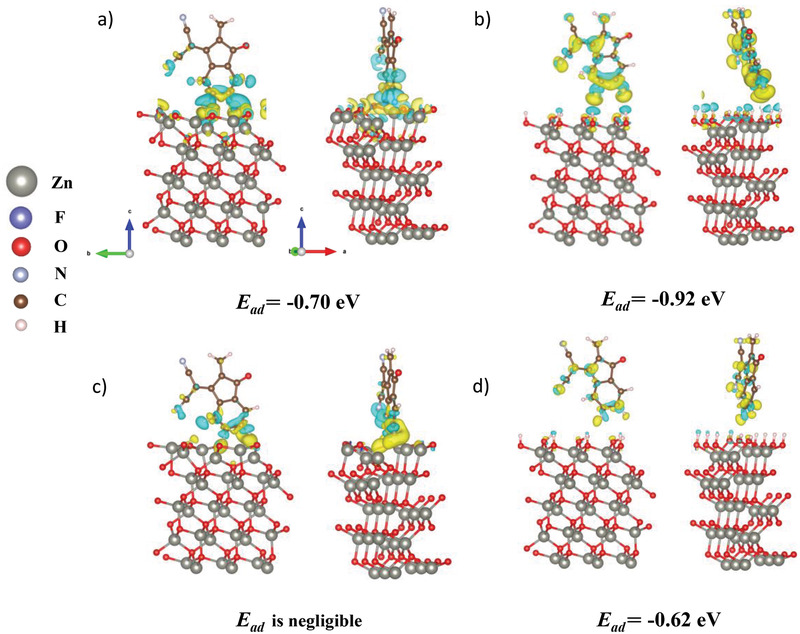
Charge density difference for NFA on ZnO(101) surface and calculated adsorption energies for the interactions of IT‐4F and ITIC with ZnO surfaces with density functional theory. The interaction between IC‐2F with a) oxygen vacancy (*V*_O_+—Zn) and b) hydroxyl group (—OH) of ZnO; the interaction between IC with c) oxygen vacancy (*V*_O_+—Zn) and d) hydroxyl group (—OH) of ZnO. Yellow/blue represents charge accumulation/depletion, where the isosurfaces refer to isovalues of 8 × 10^−4^ electrons/bohr^3^.

Our previous results demonstrated that photon‐induced formation of hydroxyl radicals on the ZnO surface is the main reactive species causing the decomposition of NFA molecules.^[^ ^25^ ^]^ A faster decomposition rate of fluorine atom‐containing acceptor molecules (IT‐4F, Y6, N3) than fluorine‐free ITIC indicates that the terminal F substitution is harmful for NFA molecules in terms of stability. To better understand the detailed mechanism, the interactions of F atoms with ZnO were investigated by comparing the adsorption energy (*E*_ad_) of 1,1‐dicyanomethylene‐3‐indanone (IC) and 5,6‐difluoro‐1,1‐dicyanomethylene‐3‐indanone (IC‐2F) on ZnO surface using a DFT calculation. ZnO (101) face was chosen as it is the most stable face for ZnO,^[^ ^33^ ^]^ and two different surface states, Zn—O—H and oxygen vacancy (*V*_O_^+^‐Zn) were selected for this simulation. **Figure** **3** shows the optimized structure of IC and IC‐2F on ZnO surface, and the *E*_ad_ of the organic moiety to different ZnO surfaces were calculated and are listed in Figure 3. As seen there, the F‐containing IC‐2F showed intensive interaction with the hydroxyl group (—OH) and oxygen vacancy (*V*_O_^+^‐Zn) through the C—F···H—O—Zn and C—F···*V*_O_^+^—Zn interaction with an *E*_ad_ of −0.92 and −0.70 eV, respectively. In comparison, the F‐free IC unit interacts only with H—O—Zn but with a lower *E*_ad_ of −0.62 eV. The more intensive interaction of F atoms with ZnO brings the NFA molecule closer to the ZnO surface, which could be one of the reasons for the faster degradation rate of the fluorine conation NFA molecules.

The influence of F atoms on the stability of NFA molecules was also investigated. The bond lengths of the C═C linker, obtained from the single crystal data,^[^ ^34^ ^]^ and the net electro population of the C═C bond, calculated by PBE0/def2TZVP method with Gaussian09 program, are found to be different among NFA molecules (Table S1, Supporting Information). IT‐4F showed a longer C═C bond (1.401 Å) and higher net electron population (−0.111) than ITIC (1.376 Å and −0.060), which is ascribed to the electron‐withdrawing effect of F atoms that pull the electron population from the central *π*‐unit (see the detailed electronic structure of the acceptors in Figure S4, Supporting Information). Such an electron‐withdrawing effect results in bond order equalization of the C—C═C bonds and leads to a longer C═C bond. The longer C═C bond length and the high electron population on the carbon atom increase the possibility of having been attacked by reactive hydroxyl radicals, which explains the lower stability of IT‐4F than ITIC (Figure 1).

There is not much difference between Y6 and N3 both for the PCE decay rate of the cells and the decomposition rate of the NFA films on ZnO (Figures 1d and 1e), indicating the alkyl chain on the central N atoms has negligible influence on the decomposition of NFA molecules on ZnO. However, the better stability of Y6/N3 than IT‐4F indicates that the core unit indeed influences the stability of the NFA molecules. In comparison with IT‐4F, Y6 showed a shorter C═C bond (1.352 Å) and a lower electron population (−0.002) on it. This can be ascribed to the electronic deficient nature of the central benzothiadiazole unit, as evidenced by the electron density distribution of the Y6 (Figure S4, Supporting Information). The shorter C═C bond and the lower electron population of the C atom in Y6 result in good accordance with the stability results shown in Figure 1.

**Figure 4:**
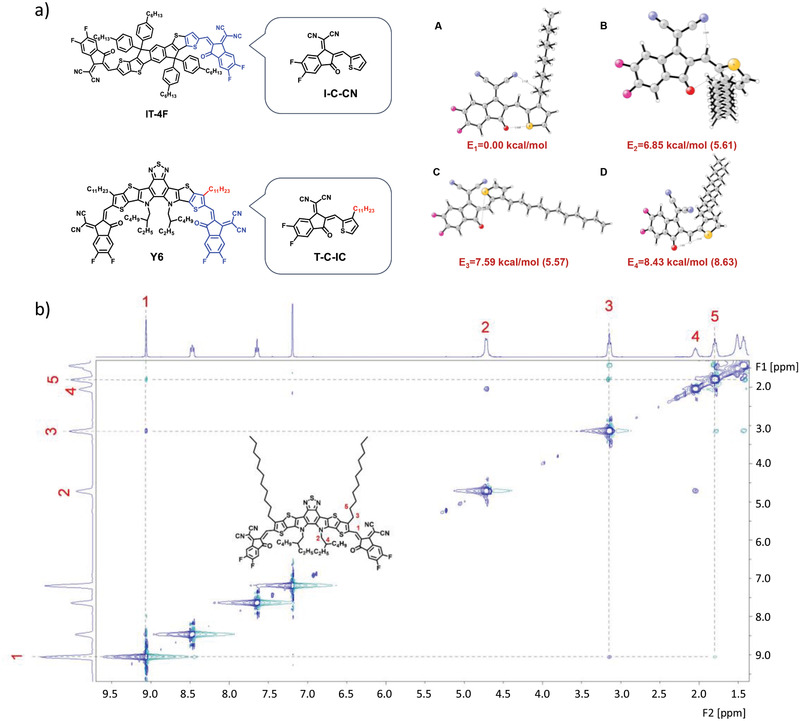
a) Diagram of torsion for I‐C‐CN and T‐C‐IC; conformation and energy level of the I‐C‐CN and T‐C‐IC calculated using DFT at the PBE0 level of theory with a basis set of def2‐SVP. The one in brackets is the energy level of the I‐C‐CN. b) ^1^H NMR NOE measurement of Y6.

Note that both Y6 and N3 have an alkyl side chain on the *β*‐position of the thiophene unit next to the C═C bond. To better understand the influence of the *β*‐alkyl side chain on the performance of the materials, we then systematically investigated the confirmation of the alkyl side chain within the molecule. Owing to the *cis*‐*trans* isomerization of the C═C bond, four different conformations can be formed. Not surprisingly, the *trans*‐confirmation showed the lowest energy (**Figure** **4**a), which is in good accordance with the crystal conformation of the Y6 and N3 molecules.^[^ ^4c^ ^]^ As seen from the simulated results, the *β*‐alkyl chain on the thiophene unit is pointing out of the conjugated plane. The ^1^H‐^1^H NOESY NMR can be used for the detection of through‐space interactions. Figure 4b shows the ^1^H NMR NOE spectrum of Y6, which confirmed intramolecular interactions among hydrogens from the aliphatic chains (—C_11_H_23_ and —C_8_H_17_) as well as the C═C bond. It can be seen from the 1D ^1^H NMR spectra and Y6 structure (inset in Figure 4b) that the H atoms from the aliphatic chains and C═C bond in different positions on Y6 have been numbered, which is convenient for discussion. The hydrogen atom on the C═C bond is numbered 1 and the hydrogen atoms on the 1‐alkyl side chain (—C_11_H_23_) are numbered 3 and 5 in sequence. Besides, the hydrogen atoms of the alkyl chain (—C_8_H_17_) on the central N atoms are numbered 2 and 4. As shown in Figure 2b strong correlation signals (dotted line junction) between hydrogen atoms 1 and 3 can be seen clearly. Further, the slightly weaker correlation signals between hydrogen atoms 1 and 5 can also be clearly observed. These results showed that hydrogen atoms on the 1‐alkyl side chain and the C═C bond showed obvious interactions. Therefore, a kind of “atom cage” forms, which was supposed to be another important protection of the C═C bond from the attacking of the reactive hydroxyl radicals. Besides, correlation signals between hydrogen atoms 2 and 4 as well as 3 and 5 can also be seen clearly. In a short summary, the *β*‐alkyl side chain plays an important role in determining the stability of the NFA molecules, which could be the direction for further development of high‐performance NFA molecules.

### 2.5 More Stable NFA Molecule with Branched *β*‐Alkyl Side Chain

**Figure 5:**
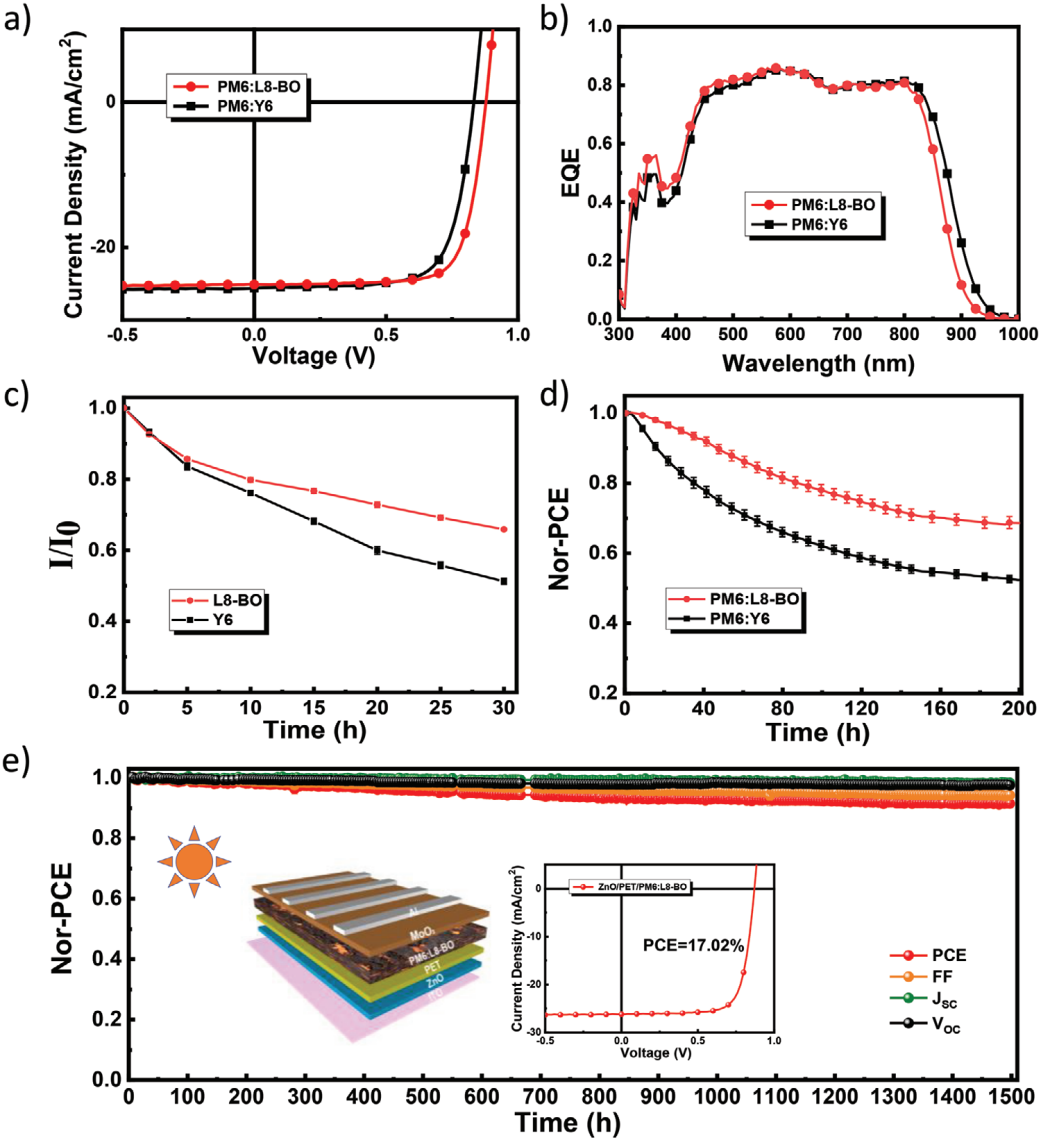
a) *J*–*V* curves and b) EQE spectra of PM6:Y6 and PM6:L8‐BO cells; c) the decrease of the absorbance of the Y6 and L8‐BO films on ZnO surface; d) PCE decay curves of PM6:Y6 and PM6:L8‐BO cells under white light illumination; and e) evolution of device performance of PM6:L8‐BO cells with surface PET treated ZnO.

After understanding the influence of molecular structure, especially the *β*‐alkyl side chain, on device stability. PSC using L8‐BO (Figure 1),^[^ ^4c^ ^]^ a new NFA molecule having branched 2‐butyloctyl chains, as the electron acceptor were fabricated and tested. **Figures** **5**a and 5b show the *J*–*V* and EQE of the best PM6:L8‐BO cell, which showed a high PCE of 16.67% with a *V*_OC_ of 0.88 V, a *J*_SC_ of 25.12 mA cm^−2^, and an FF of 0.76. For better comparison, a new set of PM6:Y6 cells were fabricated and tested. Results indicated that Y6 based cells showed a slightly lower device performance than L8‐BO (a highest PCE of 15.36% for Y6 cells, see detailed performance data in Table S2, Supporting Information). Figure S5, Supporting Information, shows the evolution of the UV–vis absorption spectra of Y6 and L8‐BO on the ZnO surface under white light illumination, and Figure 5c shows the photon bleaching rate of these two films on ZnO. Obviously L8‐BO showed a slower photon bleaching rate than Y6, which is in good accordance with the performance decay rate of the L8‐BO and Y6 based cell (see Figure 5d). This result unambiguously confirms that the *β*‐side chains next to the C═C bond have great influence on the stability of the NFA molecules and the corresponding device performance.

Our previous finding showed that treating the ZnO surface with Lewis acid, such as 2‐phenylethanethiol (PET),^[^ ^25^, ^27^ ^]^ can decrease the photon reactivity of ZnO film and consequently improve device stability. With this, a PSC with an inverted structure of ITO/ZnO (PET treated)/PM6:L8‐BO/MoO_3_/Al was fabricated, which showed the highest PCE of 17.02% (inset in Figure 5e, see also Figure S7 and Table S3, Supporting Information, for more details). The decay curves of the four device parameter (PCE, FF, J_SC_, V_OC_) are shown in Figure 5e and Figure S8, Supporting Information, is the zoom in on the decay curve of PCE of the cell. It can be seen that after a relatively fast performance decay at the beginning 800 h, the PCE decay turns to be slowed down. Even though, these cells maintained 91% of their initial PCE over 1500 h of aging (99% for *J*_SC_, 98% for *V*_OC_, and 94% for FF), indicating an excellent stability for this cell. Especially, there is almost no *J*_SC_ decay measured of the cells, indicating that the interfacial decomposition of NFA on ZnO surface was dramatically reduced with the combination of using PET‐treated ZnO and L8‐BO as the electron acceptor. Since fast *V*_OC_ and FF decays were measured for this cells, the performance decay was then ascribed to the interfacial degradation at the polymer/MoO_3_ interface.^[^ ^22^, ^23^ ^]^ Fitting the PCE decay curve of the cell to a stretched exponential model according to Equation (1) yields a stretching exponent *β* of 1, a mean lifetime (*τ*) of 971 h, and a PCE (∞) of 0.89. The mean lifetime is 6 times higher than Y6/N3 cells (Table 1), suggesting a much higher stability for the L8‐BO based cell, whereas the high PCE(∞) of 0.89 suggests that the cell should keep 89% of its initial efficiency over a long time of aging if the decay of the cell is following an exponential decay. We then fit the 2nd decay process after 800 h to a linear model, *T*_80_ and *T*s_80_, in which the time reaches 80% of its initial and stabilized efficiency,^[^ ^35^ ^]^ respectively, which were estimated to be 5140 and 6170 h by extrapolating the decay curves (Figure S10, Supporting Information). This is the first report of a high PCE of over 17% PSC with a high *T*_80_ of over 5000 h under white light illumination. It worth noting that the light source used for the aging test is UV‐free white light (see Figure S11, Supporting Information), the result reported here could not guarantee the same lifetime for the cell illuminated under a real sun condition, since intensive UV‐light is included for the sun light. However, the current work clearly demonstrates that the excellent intrinsic stability of PSC can be achieved by proper molecular structure optimization.

## 3 Conclusions

In summary, we systematically investigated the influence of NFA molecular structure on the stability of solar cells. Results showed that UV–vis absorption analyses confirmed that the PCE decay rates of these cells are directly correlated with the photobleaching process of the NFAs films on the ZnO surface, indicating that photon‐induced decomposition of NFA molecules on the ZnO surface is the main reason for the PV performance decay. The introduction of fluorine atoms on the terminal electron‐accepting moiety decreases the stability, which was mainly due to the strong intermolecular action between ZnO and fluorinated NFA molecules. More importantly, the introduction of an alkyl side chain on the *β*‐position of the thiophene unit next to the C═C bond can significantly improve the stability of the cells, which was attributed to the formation of atoms cages, which prevent the attacking of hydroxyl radical to the C═C bond. The new NFA molecule L8‐BO with a branched alkyl side chain on the *β*‐position of the thiophene unit showed improved device stability, which showed a high device performance with 17.02% and a high performance stability (with *T*_80_ over 5000 h). The current work pointed out that functionalization on the *β*‐position of the thiophene unit next to the C═C bond is the most feasible way to improve the stability of the NFA cells.

## 4 Experimental Section

### Materials

PM6 (PBDB‐T‐2F) and IT‐4F, ITIC, Y6, and N3 were purchased from Solarmer Materials Inc., Beijing. L8‐BO was purchased from Guangzhou Chasing Light Technology Co., Ltd. 2‐phenylethylmercaptan (PET), Zn(OAc)_2_, TMAH, and chlorobenzene (CB, 99.8%) were purchased from J&K Scientific Ltd. 1,8‐diiodooctane (DIO) and 1‐chloronaphthalene (CN) was purchased from sigma‐Aldrich. Molybdenum (VI) oxide (MoO_3_) was purchased from Sterm Chemicals. All materials were used as received without further purification. ZnO was prepared through the reaction of TMAH and Zn(OAc)_2_ in DMSO as reported by Qian et al.^[^ ^33^ ^]^

### Instruments and Measurement

The UV–vis absorption spectra of ZnO and NFAs films were measured with a PerkinElmer Lambada 750 at room temperature. All the films were spin‐coated on the glass substrates and aged in a glove box under white LED light. The mass spectra of NFAs were measured with matrix‐assisted laser desorption/ionization time‐of‐flight mass spectrometry (MALDI‐TOF‐MS) using *trans*‐2‐[3‐(4‐*tert*‐Butylphenyl)‐2‐methyl‐2‐propenylidene]malononitrile (DCTB) as the matrix. NFAs were spin‐coated on the glass substrates and degraded in white LED light for 5 and 10 h. Then the degraded films were dissolved with methylene chloride (CH_2_Cl_2_) and dried under vacuum. ^1^H NMR spectra were recorded on a Bruker Ascend 400 MHZ NMR spectrometer at room temperature with deuterated chloroform as the solvent.

### Fabrication of Polymer Solar Cells

ITO substrates were sequentially cleaned by detergent, deionized water, acetone, and isopropanol in an ultrasound cleaner. Then they were put in the isopropanol for storage. Before using them, they were first dried by N_2_ flow and then treated in a UV–ozone oven for 30 min. First, ZnO NPs (10 mg mL^−1^ in ethanol) was spin‐coated on the ITO substrates at 2000 rpm for 60 s and then were annealed at 130 °C for 10 min on a hot plate in a glove box filled with N_2_. The solution of PM6:IT‐4F and PM6:ITIC (10 mg mL^−1^ for each compound) blended in chlorobenzene (CB) with 0.5 vol% DIO was spin‐coated on the top of the ZnO electron transportation layer at 2000 rpm for 60 s and then were annealed at 130 °C for 10 min on a hot plate in a glove box filled with N_2_. The mixed solution of PM6:Y6 together with 0.5 vol% CN was dissolved in CF with concentrations of 7 and 8.4 mg mL^−1^, respectively. The blend solution of PM6:L8‐BO was prepared by dissolving PM6 and L8‐BO in chloroform solvent with 0.5 vol% CN. The total concentration of PM6:L8‐BO solution was maintained at 15.4 mg mL^−1^. Finally, MoO_3_ (20 nm) as the hole‐extraction layer and Al (100 nm) as the anode were sequentially vacuum deposited on the top of the active layer respectively. The effective photovoltaic area, defined by the geometrical overlap between the bottom cathode electrode and the top anode, was 0.09 cm^2^. Note that the performance decay of the PM6:IT‐4F cells was reported in our previous paper already.^[^ ^25^ ^]^ For better comparison, however, we fabricated a new set of the PM6:IT‐4F cells and tested the decay dynamics simultaneously with other cells.

### PV Parameters of Polymer Solar Cells

The PV parameters of the cells including *V*_OC_, *J*_SC_, and FF were measured using a Keithley 2400 source meter under illumination with simulated AM 1.5G sunlight (Zolix, Sirius‐SS150A) in a glove box filled with N_2_. The EQE spectra were recorded by EQE system which was built in home and the light from a 150 W tungsten halogen lamp (Osram 64 610) was used as a probe light and was modulated with a mechanical chopper before passing through the monochromator (Zolix, Omni‐k300) to select the wavelength. The response was recorded as the voltage by an *I*–*V* converter (D&R‐IV Converter, Suzhou D&R Instruments), using a lock‐in amplifier (Stanford Research Systems SR 830) with a stand silicon cell as the reference before testing the devices.

### Theoretical Calculation

The single crystal structure data used in the calculation comes from CCDC (https://www.ccdc.cam.ac.uk/).

### Degradation of Polymer Solar Cells under White Light

The long‐term stability of un‐encapsulated devices was conducted by multi‐channel solar cell performance decay test system (PVLT‐G8001M, Suzhou D&R Instruments Co. Ltd.) under a testing condition in accordance with ISOS‐L‐1 in the glove box. The cells were put inside a glove box filled with N_2_ (H_2_O <10 ppm, O_2_ <10 ppm) and continuously illuminated with white LED light (D&R Light, L‐W5300KA‐150, Suzhou D&R Instruments, see Figure S11, Supporting Information, for the spectrum). The illumination light intensity was initially set so the output short‐circuit current density (*J*_SC_) is as same as that measured under standard conditions by AM 1.5G. For monitoring changes in illumination light intensity, it was monitored by a photodiode (Hamamtsu S1336‐8BQ). *J*–*V* characters of the devices were checked periodically, and the photovoltaic performances data (*V*_OC_, *J*_SC_, FF, and PCE) were calculated automatically according to the *J*–*V* curves. In between the *J*–*V* tests, an external resistor load matching the maximum power output point (*R*_mpp_ = *V*_max_/*I*_max_), was attached to the cell. The external load can dynamically change according to the *J*–*V* results, the measured performance decay curves is therefore close to the decay behaviors of the cells under a real operation. The stability test method of using passive resistor fits the highest recommended testing protocol ISOS‐L3.^[^ ^36^ ^]^The temperature of the cells is thermostatically controlled at room temperature 25 °C by temperature control equipment.

## Conflict of Interest

The authors declare no conflict of interest.

## Supporting information

Supporting Information

## Data Availability

Research data are not shared.
