# Supplementary material for: Simultaneously Achieving Highly Efficient and Stable Polymer:Non‐Fullerene Solar Cells Enabled By Molecular Structure Optimization and Surface Passivation
Source: Adv Sci (Weinh). 2022 Jan 15;9(7):2104588. doi: 10.1002/advs.202104588 (PMC8895120; doi:10.1002/advs.202104588)
Supplement: Supplementary file 1 — Supporting Information [file ADVS-9-2104588-s001.pdf]

ADVANCED SCIENCE
Open Access

Supporting Information

for *Adv. Sci.,* DOI: 10.1002/advs.202104588

Simultaneously achieving highly efficient and stable polymer:non-fullerene solar cells enabled by molecular structure optimization and surface passivation

Bowen Liu, Xiao Su, Yi Lin, Zerui Li, Lingpeng Yan, Yunfei Han, Qun Luo, Jin Fang, Shangfeng Yang, Hongwei Tan,* Chang-Qi Ma**

Supporting Information:

Simultaneously achieving highly efficient and stable polymer:non-fullerene solar cells enabled by molecular structure optimization and surface passivation

Bowen Liu,[a,b] Xiao Su [c], Yi Lin,[d] Zerui Li [a,b] Lingpeng Yan,[b] Yunfei Han [a,b] Qun Luo,[a,b]* Jin Fang,[b] Shangfeng Yang,[e] Hongwei Tan,[c]* Chang-Qi Ma[a,b]*

[a] School of Nano-Tech and Nano-Bionics, University of Science and Technology of China, Hefei, 230026, P. R. China

[b] i-Lab, Suzhou Institute of Nano-Tech and Nano-Bionics, Chinese Academy of Sciences, Ruoshui Road 398, SEID, SIP, 215123, P. R. China

[c] College of Chemistry, Beijing Normal University, Xinjiekouwai St. Beijing 100875 P.R.China

[d] Department of Chemistry, Xi'an Jiaotong Liverpool University, Renai Road 11, SEDI, SIP, Suzhou, 215123, P. R. China

[e]CAS Key Laboratory of Materials for Energy Conversion, Department of Materials Science and Engineering, University of Science and Technology of China, Hefei 230026, P. R. China

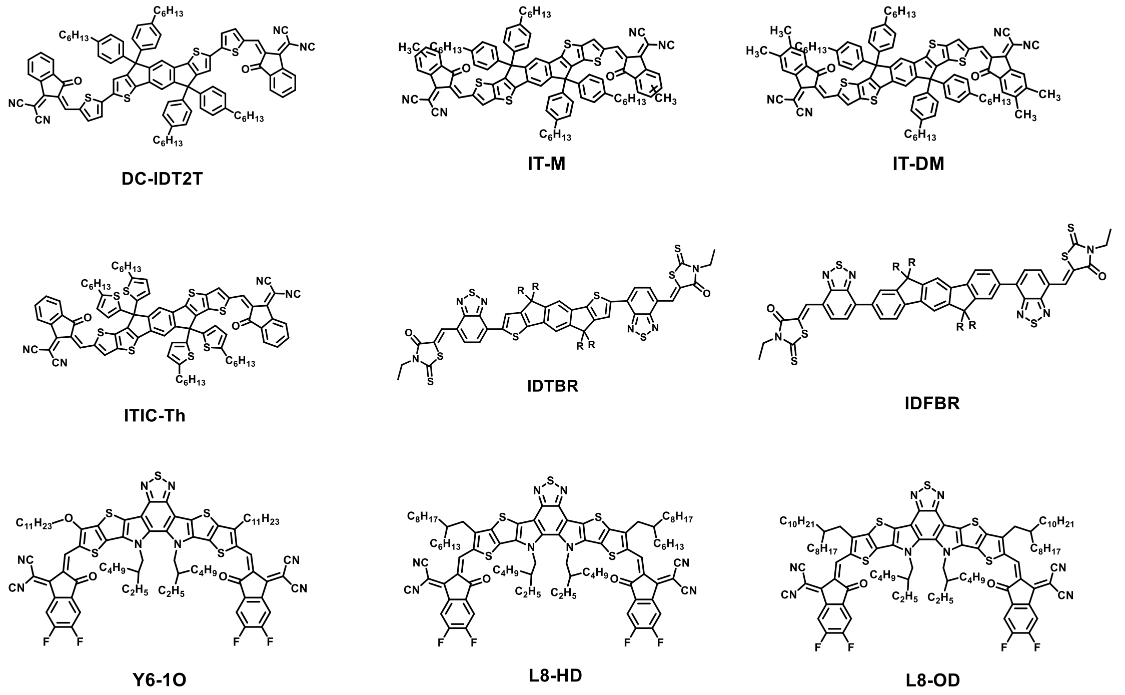

Scheme S1. Chemical structure of the NFA molecules reported in the literatures

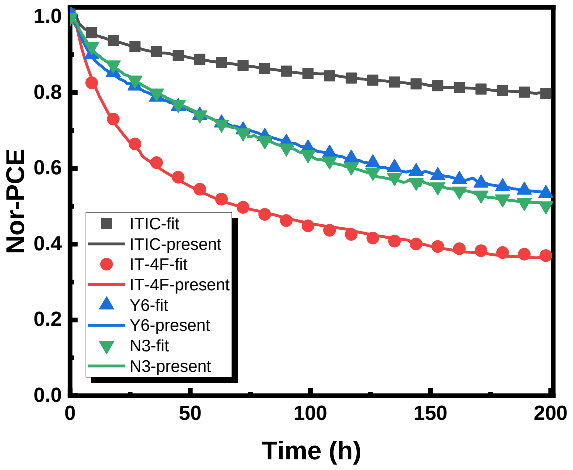

Figure S1. Fiting the PCE decays of the PM6:NFA cells to a streched exponatial decay model

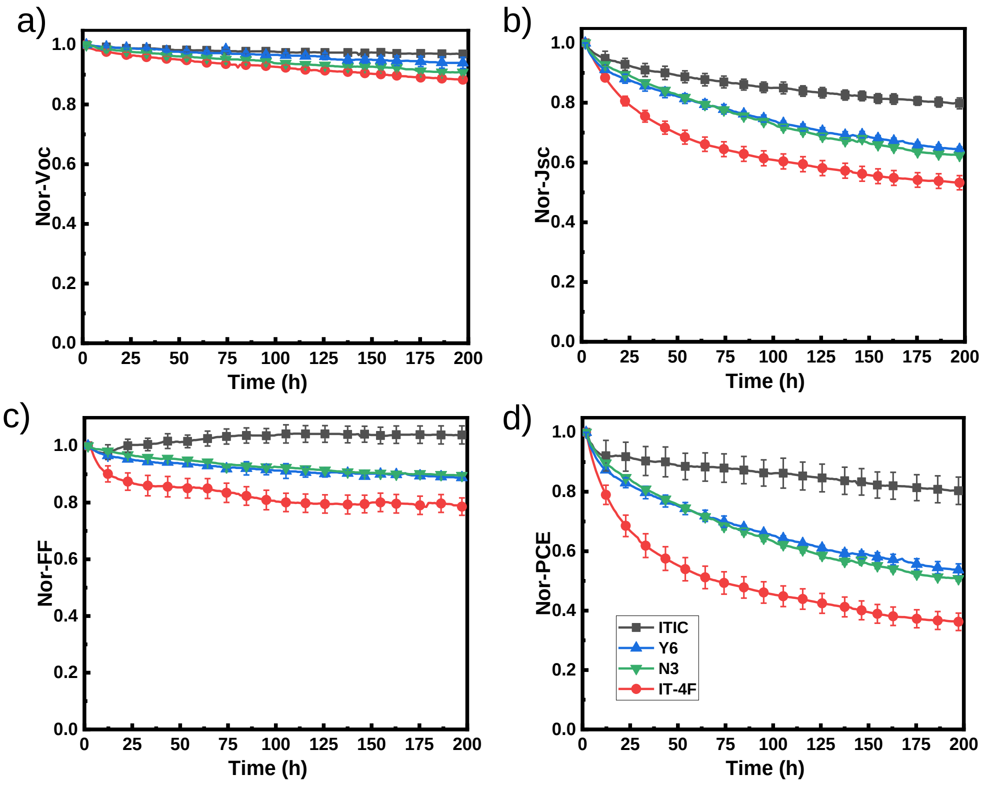

Figure S2. PCE decay curves of PM6:NFAs cells under white light illumination

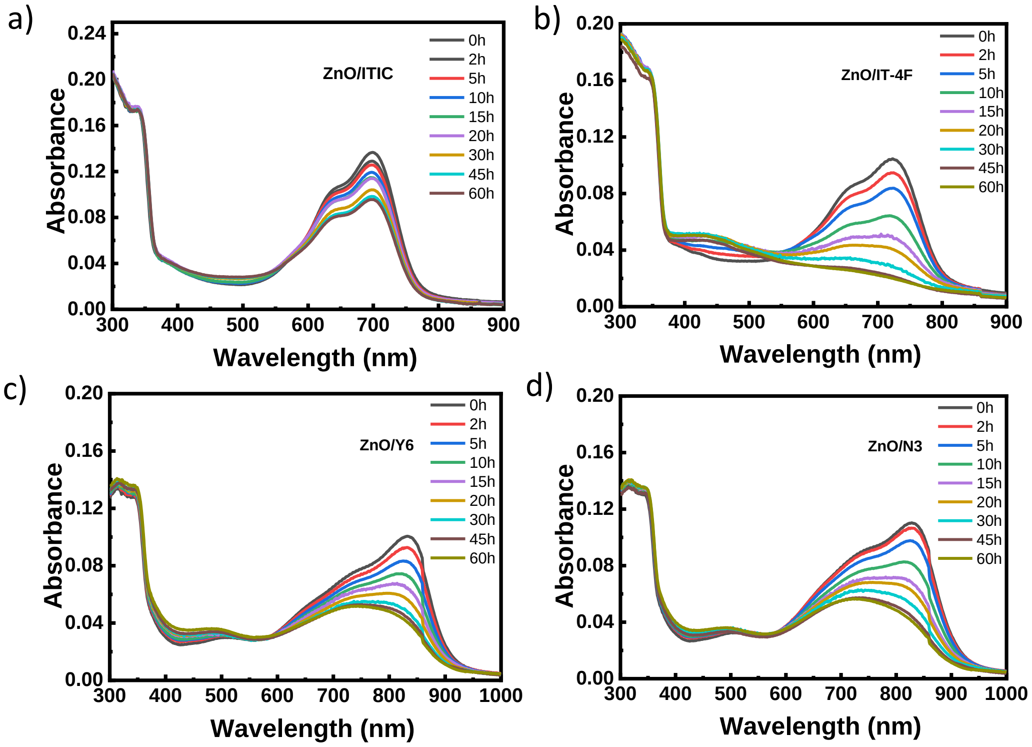

Figure S3. UV-vis absorption changes a): ZnO/ITIC; b): ZnO/IT-4F; c): ZnO/Y6; d): ZnO/N3 under white light illumination

Table S1. Bond length and electronic density of the C=C bond

Compound	CCDC number	C=C bond length (Å)[a]	Net Electron Population		
			C[b]	C[c]	C=C
ITIC	1885952	1.376	-0.060	0.018	-0.042
IT-4F	1885953	1.401	-0.111	0.016	-0.094
Y6	2006203	1.352	-0.002	-0.030	-0.032

a: bond length obtained from the single crystal structure; b: the C atom linked to the thiophene ring; c: C atom on the five member ring

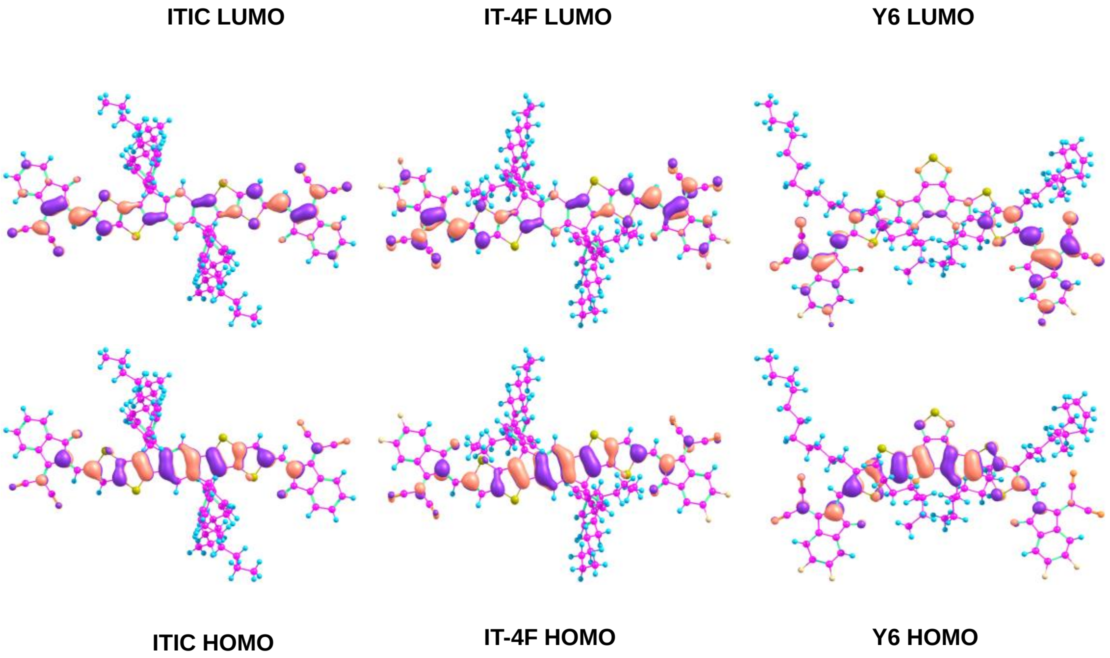

Figure S4. Electronic structure of ITIC, IT-4F, Y6

Table S2. Performance of devices based on PM6:Y6 and PM6:L8-BO[a]

ATL	V_{oc} (V)	J_{sc} (mA/cm^2)	FF	PCE (%)	PCE_{max} (%)[d]
**PM6:Y6[b]**	**0.83±0.002**	**25.05±0.372**	**0.72±0.003**	**14.96±0.284**	**15.36**
**PM6:L8-BO[c]**	**0.87±0.006**	**25.02±0.097**	**0.76±0.002**	**16.47±0.219**	**16.67**

a: device structure ITO/ZnO/PM6:NFA/MoO_3/Al, cell area 0.09 cm^2, averaged device performance over 8 individual cells; b: blend ratio PM6:Y6 (1.0:1.2 in weight) with Chloronaphthalene (CN) as an additive ; c: blend ratio PM6:L8-BO (1.0:1.2 in weight) with CN as an additive; d: PCE of the best cell.

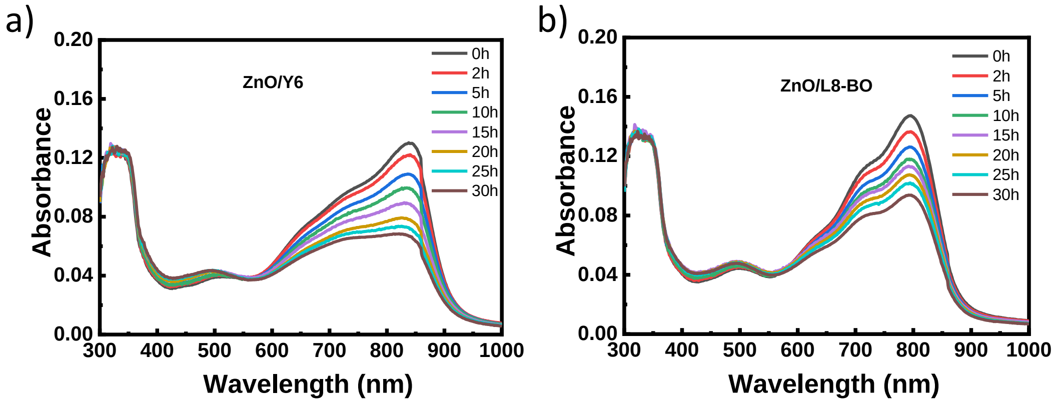

Figure S5. UV-vis absorption changes a): ZnO/Y6; b): ZnO/L8-BO under light illumination inside the glovebox

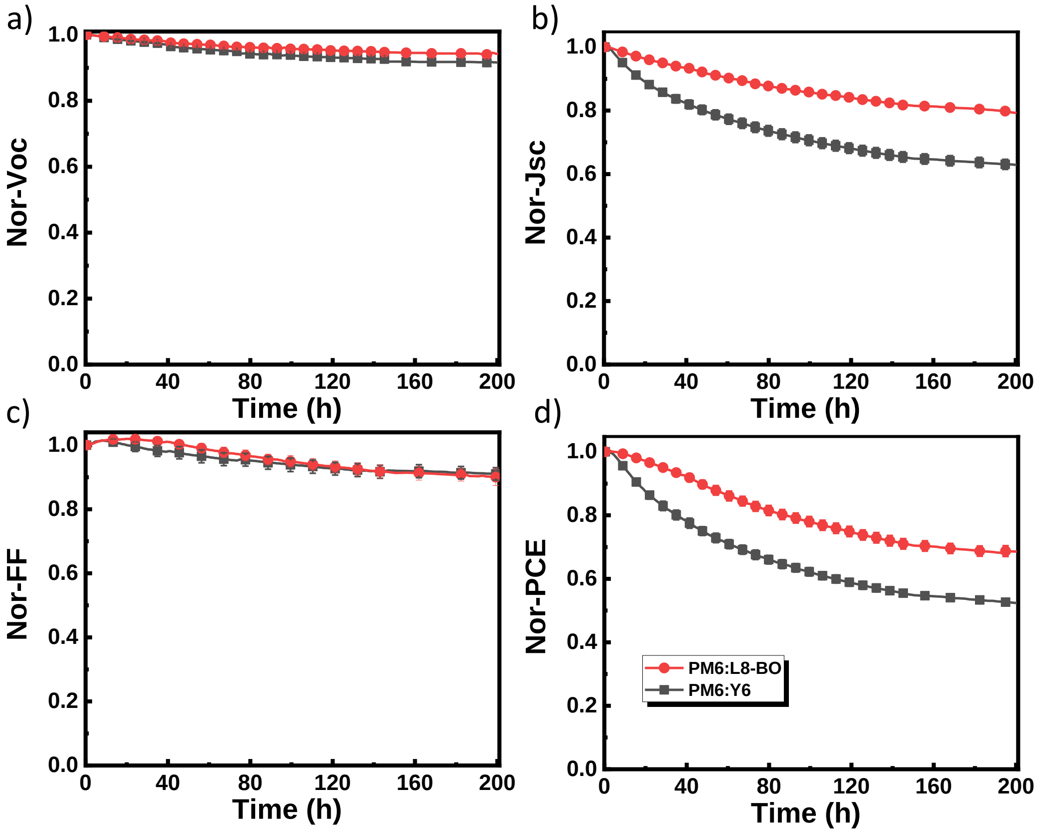

Figure S6. PCE decay curves of PM6:Y6 and PM6:L8-BO cells under white light illumination

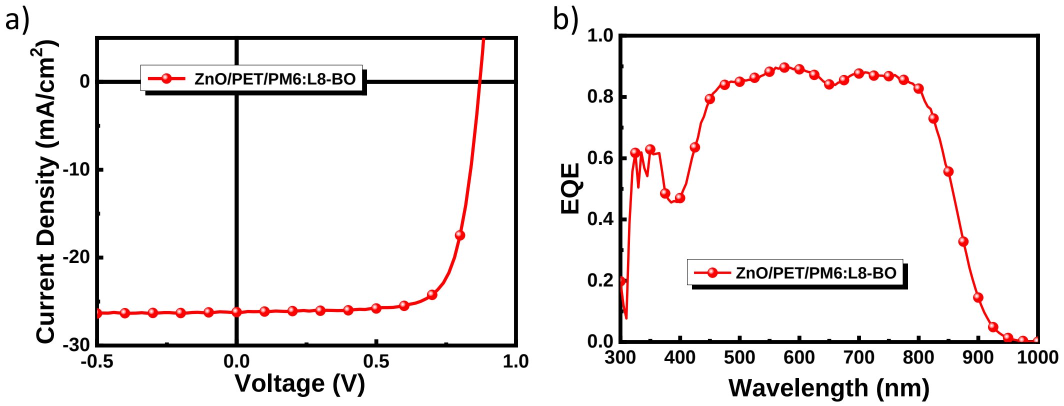

Figure S7. a) *J-V* curves and b) EQE spectra of ZnO/PET/**PM6**:**L8-BO** cell

Table S3. Performance of devices based on ZnO/PET/PM6:L8-BO[a]

ETL	V_{oc} (V)	J_{sc} (mA/cm^2)	FF	PCE (%)	PCE_{max} (%) [d]
**ZnO/PET**	**0.87±0.003**	**26.05±0.181**	**0.75±0.003**	**16.94±0.098**	**17.02**

a: device structure ITO/ZnO/PET/PM6:L8-BO/MoO_3/Al, cell area 0.09 cm^2, averaged device performance over 8 individual cells

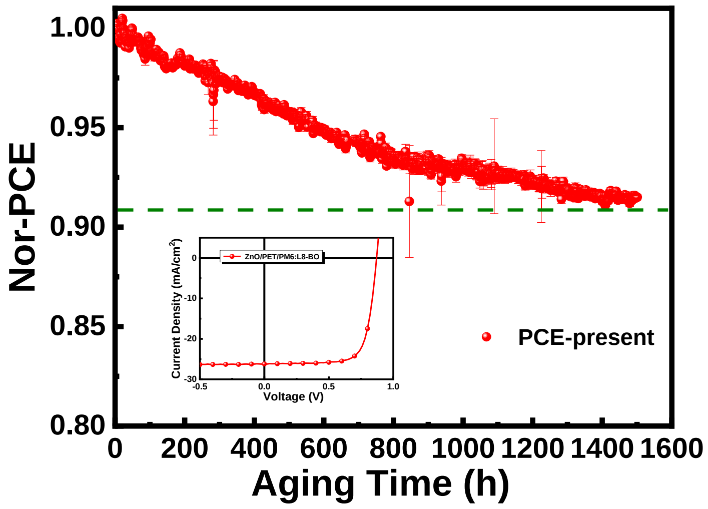

Figure S8. PCE decay curves of ITO/ZnO/PET/ PM6:L8-BO/MoO_3/Al cells under white light illumination

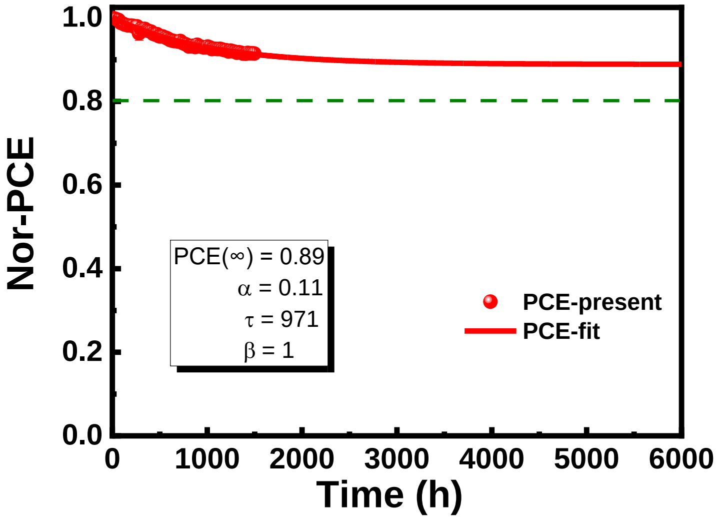

Figure S9. PCE decay curves of ITO/ZnO/PET/ PM6:L8-BO/MoO_3/Al cells under white light illumination and the PCE decays were numerically fitted to a stretched exponential model according to the equation (1)

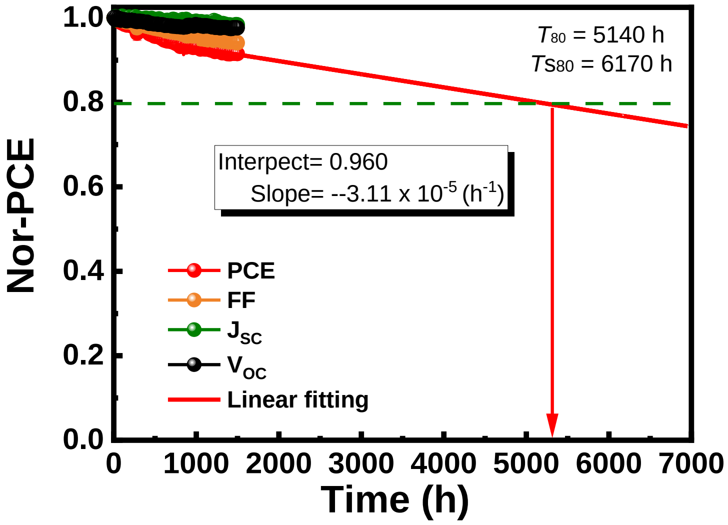

Figure S10. Decay curves of ITO/ZnO/PET/ PM6:L8-BO/MoO_3/Al cells under white light illumination. T_{80} and Ts_{80} were estimated to be 5140 and 6170 hours, respectively, by extrapolating the second decay process (after 800 hours)

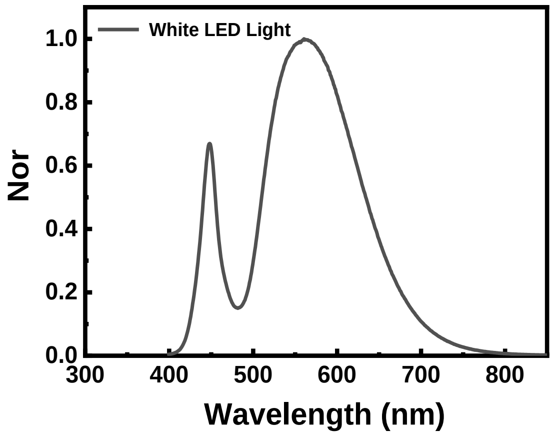

Figure S11. Light spectrum of the white LED light used in manuscript.
